# *In vivo* and *in vitro* mutagenicity of perillaldehyde and cinnamaldehyde

**DOI:** 10.1186/s41021-021-00204-3

**Published:** 2021-07-16

**Authors:** Masamitsu Honma, Masami Yamada, Manabu Yasui, Katsuyoshi Horibata, Kei-ichi Sugiyama, Kenichi Masumura

**Affiliations:** 1 Division of Genetics and Mutagenesis, National Institute of Health Sciences, 3-2-26 Tonomachi, Kawasaki City, Kanagawa 210-9501 Japan; 2 Division of General Affairs, National Institute of Health Sciences, Kawasaki City, Japan; 3 Department of Applied Chemistry, National Defense Academy, Yokosuka City, Japan

**Keywords:** Perillaldehyde, Cinnamaldehyde, Mutagenicity, Quantitative structure–activity relationship, Ames test, Transgenic rodent gene mutation assay

## Abstract

**Background:**

Perillaldehyde and cinnamaldehyde are natural substances found in plants that are used as flavoring ingredients. Due to the α,β-unsaturated aldehydes in their structures, these compounds are expected to be DNA reactive. Indeed, several reports have indicated that perillaldehyde and cinnamaldehyde show positive in in vitro and in vivo genotoxicity tests. However, their genotoxic potentials are currently disputed. To clarify the mutagenicity of perillaldehyde and cinnamaldehyde, we conducted in silico quantitative structure–activity relationship (QSAR) analysis, in vitro Ames tests, and in vivo transgenic rodent gene mutation (TGR) assays.

**Results:**

In Ames tests, perillaldehyde was negative and cinnamaldehyde was positive; these respective results were supported by QSAR analysis. In TGR assays, we treated Muta™ Mice with perillaldehyde and gpt-delta mice with cinnamaldehyde up to the maximum tested doses (1000 mg/kg/day). There was no increase in gene mutations in the liver, glandular stomach, or small intestine following all treatments except the positive control (N-ethyl-N-nitrosourea at 100 mg/kg/day).

**Conclusions:**

These data clearly show no evidence of in vivo mutagenic potentials of perillaldehyde and cinnamaldehyde (administered up to 1000 mg/kg/day) in mice; however, cinnamaldehyde is mutagenic in vitro.

## Introduction

Perillaldehyde (Table 1) is a natural substance found abundantly in the plant *Perilla frutescens* (Shiso in Japanese) from the mint family Lamiaceae. Because perillaldehyde has strong antiseptic and bactericidal actions and its scent produces an appetite-enhancing effect [1, 2], Shiso is used as an accessory herb when eating raw fish (sashimi) and as an essential ingredient of salted plum (umeboshi) in Japan. In addition, perillaldehyde is used as a flavoring ingredient for salad dressings, sauces, pickled vegetables, and beverages.

**Table 1:**
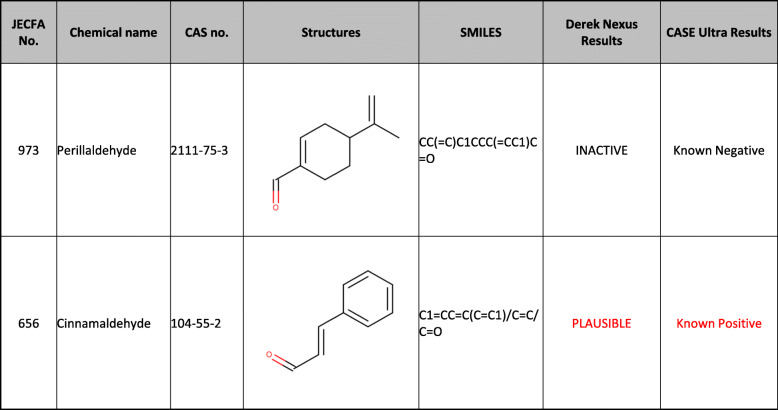
The results of (Q) SAR prediction for Ames mutagenicity of perillaldehyde and cinnamaldehyde

Cinnamaldehyde (Table 1) is one of the aromatic aldehydes contained in cinnamon. Its main uses are as a flavoring for chewing gum, ice cream, candies, and beverages. It is also used as a fragrance in cosmetics, soaps, and detergents. Cinnamaldehyde is often used as a stomachic, an antipyretic, and an antiallergic drug or as a tonic in traditional Chinese medicines [3]. Furthermore, the antifungal and antibacterial effects of cinnamaldehyde can help reduce infections [4, 5].

In terms of their safety, perillaldehyde and cinnamaldehyde have been “generally recognized as safe” (GRAS) by the Expert Panel of the U.S. Flavor and Extract Manufactures Association (FEMA), they have been approved for use by the Food and Drug Administration of the United States, and they were judged to be safe by the Food and Agriculture Organization of the United Nations/World Health Organization Joint Expert Committee on Food Additives [6, 7]. On the other hand, the genotoxicity of perillaldehyde and cinnamaldehyde are potential concerns due to the presence of α and β-unsaturated aldehydes in their structures; these unsaturated aldehydes are electrophilic and can react with electron-rich macromolecules, including DNA, to form DNA adducts [8]. Indeed, previous reports have indicated that perillaldehyde and cinnamaldehyde showed positive results in vitro or in vivo genotoxicity tests [9–11].

Of the many types of genotoxicity, mutagenicity is an important mechanism of chemical-mediated carcinogenesis that is based on the reactivity between DNA and chemical substances resulting in mutations [12]. Given that mutations are irreversible and permanent, mutagenicity does not have a threshold because just one mutation in the genome has the potential to generate a cancerous cell. If a chemical is mutagenic, the risk of cancer cannot be zero, even at low dosage levels [13]. Therefore, determining the presence or absence of a chemical substance’s mutagenicity is an important step in cancer risk assessment.

In the present study, the mutagenicity of perillaldehyde and cinnamaldehyde were determined systematically using in silico quantitative structure–activity relationship analysis (QSAR), in vitro Ames tests, and in vivo transgenic rodent gene mutation (TGR) assays.

## Materials and methods

### Test chemicals

Perillaldehyde (CAS no. 2111-75-3, lot no. 180823, 97.3% purity; manufactured by Nippon Terpene Chemicals, Inc., Kobe, Japan) and cinnamaldehyde (CAS no. 14371–10-9, lot no. 8602102, 99.7% purity; manufactured by Inoue Perfumery MFG. Co., Ltd., Tokyo, Japan) were supplied through Japan Flavor & Fragrance Materials Association.

### QSAR analysis

We used both rule- and statistical-based QSAR tools [14]. Derek Nexus 6.1.0 is a rule-based expert SAR system developed by Lhasa Limited, UK [15, 16]. The knowledge base includes structural alerts for Ames mutagenicity that have been implemented by experts who assessed relevant Ames data and supporting mechanistic data (e.g., DNA adduct-formation experiments). When a query compound matches a structural alert, Derek Nexus offers the relevant inference level (e.g., certain, probable, plausible, equivocal, doubted, or improbable), which indicates the likelihood that compounds in a class will be active in an Ames test. In our tests, a positive prediction was assigned to the query compound when the reasoning level was equivocal or above.

CASE Ultra is statistical-based QSAR software developed by MultiCASE Inc. (USA). It uses a statistical method to automatically extract alerts based on training data via machine learning technology [17, 18]. In this study, we used CASE Ultra version 1.8.0.2 with the GT1_BMUT module. The prediction result of each module was ranked as “known positive,” “positive,” “negative,” “known negative,” “inconclusive,” or “out of domain.” A query chemical that ranked as “known positive,” “positive,” or “inconclusive” in the Ames test was predicted to be positive.

### Ames test

Using the preincubation method, Ames tests were conducted by contract research organizations following Good Laboratory Practice (GLP) compliance according to the Industrial Safety and Health Act test guidelines [19]. The test guidelines require the use of five strains (*Salmonella thyphimurium* TA100, TA98, TA1535, and TA1537, and *Escherichia coli* WP2 uvrA) under both the presence and absence of metabolic activation (rat S9-mix), which is similar to the Organization of Economic Co-operation and Development (OECD) guideline TG471 [20]. The positive criterion was the number of revertant colonies increasing by more than two-fold the control in at least one Ames test strain in the presence or absence of S9-mix. Dose dependency and reproducibility were also considered in the final judgment.

### TGR assay

TGR assays were conducted by contract research organizations following GLP compliance according to the OECD guideline TG488 [21]. Animals were treated in accordance with regulations of the Animal Care and Use Committees of the laboratories and the National Institute of Health Sciences, Japan.

#### TGR assay for perillaldehyde using Muta™ mice

Male Muta™ Mice (CD_2_-LacZ80/HazfBR) were purchased at 8 weeks of age from Japan Laboratory Animals, Inc. (Tokyo, Japan). Administration of perillaldehyde started at 9 weeks of age. Six mice each were treated with perillaldehyde at 125, 250, 500, or 1000 mg/kg/day by oral gavage for 28 days, with corn oil used as a vehicle. Three days after the final treatment, the liver and glandular stomach were each collected and stored. As a positive control group, mice were treated with N-ethyl-N-nitrosourea (ENU) at 100 mg/kg/day by intraperitoneal (i.p.) injection for two consecutive days. Genomic DNA was extracted from the liver and glandular stomach (whole tissue) using the phenol/chloroform method. Transgenes were rescued via an in vitro packaging reaction using Transpack Packaging Extracts (Agilent Technologies, CA). Mutant frequency (MF) was estimated via the *lacZ* positive selection method [22]. Five mice each from the highest three dose groups (i.e., 250, 500, and 1000 mg/kg/day) were used for mutation assays. MFs were statistically analyzed using Dunnett’s test to compare treated groups against the vehicle control group and using Student’s or Welch’s t-test to compare the positive control group against the vehicle control. A significance level of 5% was adopted with two-tailed tests.

#### TGR assay for cinnamaldehyde using *gpt* delta mice

Male C57BL/6 J *gpt* delta transgenic mice (C57BL/6JJmsSlc-Tg) were purchased at 6 weeks of age from Japan SLC, Inc. (Shizuoka, Japan). Administration of cinnamaldehyde started at 7 weeks of age. Seven to ten mice each were treated with cinnamaldehyde at 125, 250, 500, and 1000 mg/kg/day by oral gavage for 28 days with corn oil as the vehicle. Three days after the final treatment, the liver and small intestine were again each collected and stored. As a positive control, previously collected bone marrow DNA was used. The positive control DNA for the *gpt* assay was extracted from ENU (50 mg/kg/day, i.p., 5 consecutive days)-treated mice sacrificed 14 days after the final treatment. The positive control DNA for the *Spi*^*−*^ assay was extracted from mitomycin C (1 mg/kg/day, i.p., 5 consecutive days)-treated mice sacrificed 7 days after the final treatment. Genomic DNAs of the liver and small intestine (whole tissue) were extracted using a RecoverEase DNA Isolation Kit (Agilent Technologies). Transgenes were rescued by an in vitro packaging reaction using Transpack Packaging Extracts. MF was estimated by the *gpt* assay for point mutations and by the *Spi*^*−*^ assay for deletions [23]. Five animals each from the two highest dose groups (500 and 1000 mg/kg/day) and three animals each from the positive control groups were used for the mutation assays. MFs were statistically analyzed by one-tailed Dunnett’s tests or Steel tests with a significance level of 5% to compared treated groups against the vehicle control. One-tailed Student’s or Welch’s t-tests were used to compare the positive control against the vehicle control at a significance level of 5%.

## Results

### QSAR analysis

We used two QSAR tools (DEREK Nexus and CASE Ultra) to predict the Ames mutagenicity of perillaldehyde and cinnamaldehyde. Perillaldehyde was judged negative for mutagenicity by both the QSAR tools, whereas cinnamaldehyde was judged positive by both (Table 1).

### Ames tests

Following treatment with perillaldehyde, the number of revertant colonies did not increase in any of the strains in the presence or absence of S9-mix; however, cytotoxicity was observed from 313 μg/plate in all treatments (Table 2). On the other hand, cinnamaldehyde treatment dose-dependently induced revertant colonies in TA100 in both the presence and absence of S9-mix (Table 3). However, the maximum number of revertant colonies was 213 (TA100 in the absence of S9-mix) at 313 μg/plate, which was slightly more than twice the number (105 colonies) detected in the negative control (DMSO) and indicates weak mutagenicity. Signs of cytotoxicity were observed at 625 μg/plate in all cinnamaldehyde treatments. According to these results, we concluded that perillaldehyde was negative and cinnamaldehyde was positive in the Ames tests.

**Table 2:**
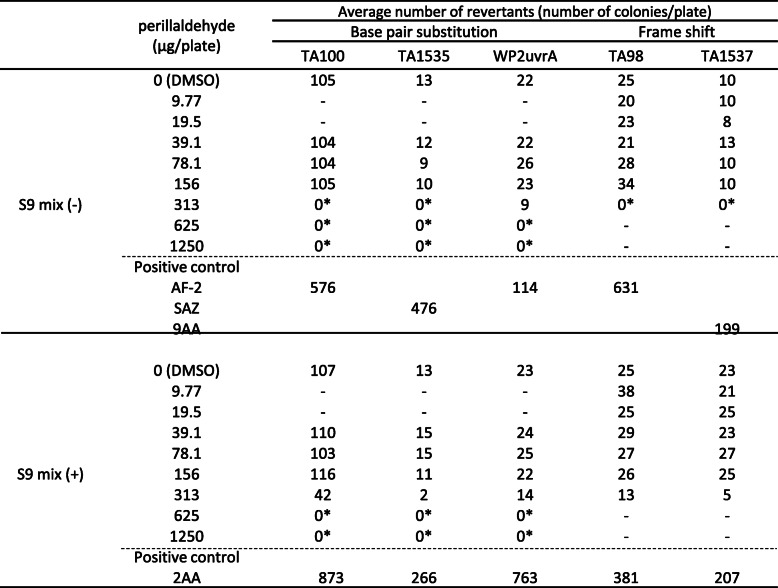
The results of the Ames test with perillaldehyde

Abbreviations: AF-2: 2-(2-Furyl)-3-(5-nitro-2-furyl) acrylamide, SA: Sodium azide, 9AA: 9-Aminoacridine, B [a]P:

Benzo [a] pyrene, 2AA: 2-Aminoanthracene, NT: Not tested

Asterisks (*) represent that growth inhibition was observed

**Table 3:**
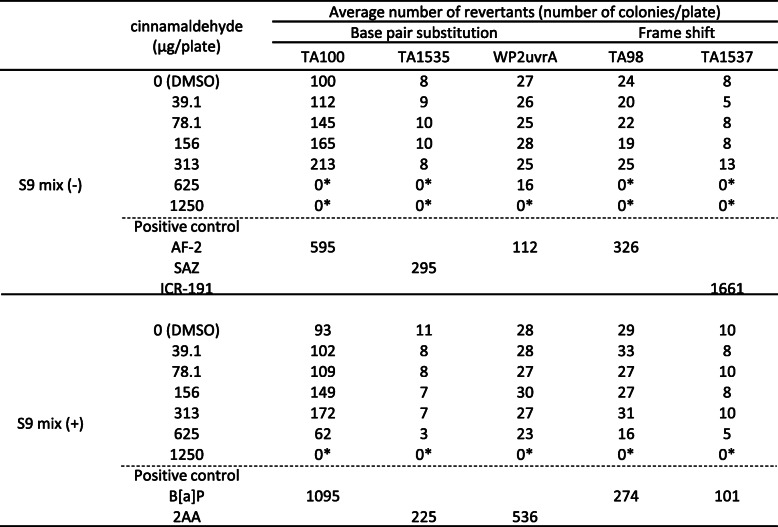
The results of the Ames test with cinnamaldehyde

Abbreviations: AF-2: 2-(2-Furyl)-3-(5-nitro-2-furyl) acrylamide, SA: Sodium azide, 9AA: 9-Aminoacridine, B [a]P:

Benzo [a] pyrene, 2AA: 2-Aminoanthracene, NT: Not tested

Asterisks (*) represent that growth inhibition was observed

### TGR assays

#### Perillaldehyde treatment in Muta™ mice

In the 1000-mg/kg/day perillaldehyde treatment group, one death was observed on day 5 (before the treatment). However, no significant weight loss was observed in all cases (except for the dead mouse; data not shown). MFs of *lacZ* genes in the liver and glandular stomach tissues from perillaldehyde-treated mice were not significantly higher than MFs of genes in comparable tissues from negative control animals (Tables 4 and 5). In contrast, the positive control ENU significantly increased MFs in the liver and glandular stomach (*p* ≤ 0.05).

**Table 4:**
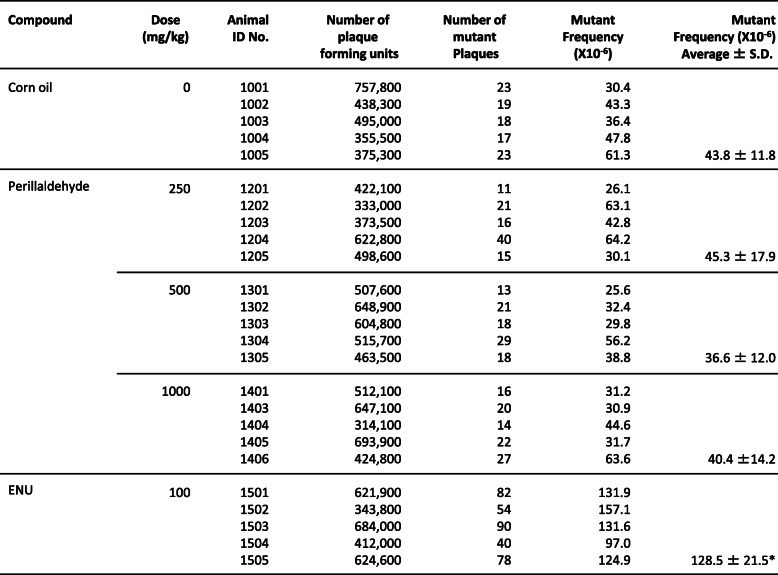
The results of TGR assay in liver of MutaTM Mouse after perillaldehyde treatment

Corn oil: Negative control (5 mL/kg)

ENU: positive control (N-ethyl-N-nitrosourea, 10 mL/kg*, i.p.*, dose once a day, for 2 days, expression period; 10 days)

*p < 0.05, significant difference from control (Kastenbaum and Bowman method, upper-trailed)

**Table 5:**
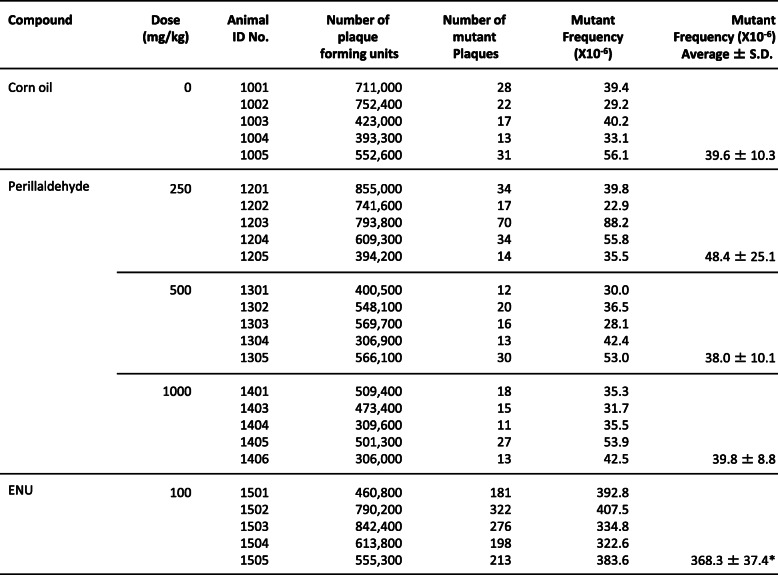
The results of TGR assay in glandular stomach of MutaTM Mouse after perillaldehyde treatment

Corn oil: Negative control (5 mL/kg)

ENU: positive control (N-ethyl-N-nitrosourea, 10 mL/kg*, i.p.*, dose once a day, for 2 days, expression period; 10 days)

*p < 0.05, significant difference from control (Kastenbaum and Bowman method, upper-trailed)

#### Cinnamaldehyde treatment in *gpt* delta mice

During the treatment of cinnamaldehyde, one death in 7 animals was observed in each of 250 mg/kg/day (on day 6) and 500 mg/kg/day treatment group (on day 5). In the 1000-mg/kg/day treatment group, four death in 10 animals were observed (on day 5, 5, 7, 14). No significant weight loss was observed in other mice. Gene mutation analysis was performed in the highest dosage groups (500 and 1000 mg/kg/day); no significant increase in MF was observed in cinnamaldehyde-treated mice tested in th*e gpt* assay of their liver and small intestine (Tables 6 and 7). In *Spi-* assays of the liver and small intestine, a significant increase in MF was not detected in cinnamaldehyde-treated groups (data not shown). On the other hand, *gpt* and *Spi* MFs in the positive control groups (ENU and MMC, respectively) showed significant increases at the 5 and 1% significance levels, which confirm the validity of this study system.

**Table 6:**
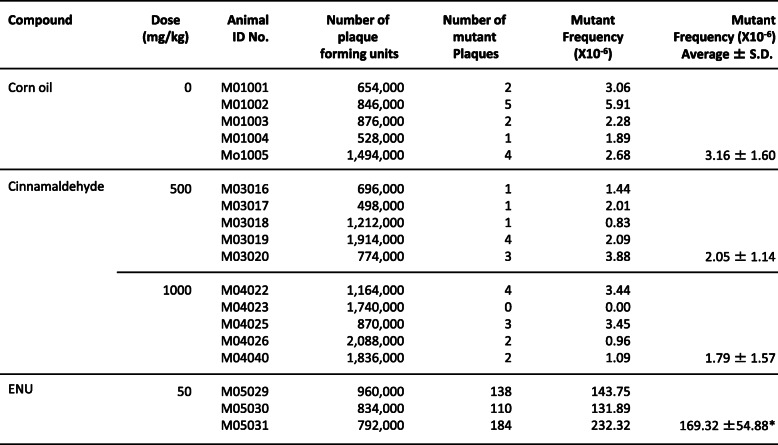
The results of TGR assay in liver of gpt delta mice after cinnamaldehyde treatment

Corn oil: Negative control (5 mL/kg)

ENU: positive control (N-ethyl-N-nitrosourea, 10 mL/kg*, i.p.*, dose once a day, for 5 days, expression period; 10 days)

*p < 0.05, significant difference from control (Welch’s I-test)

**Table 7:**
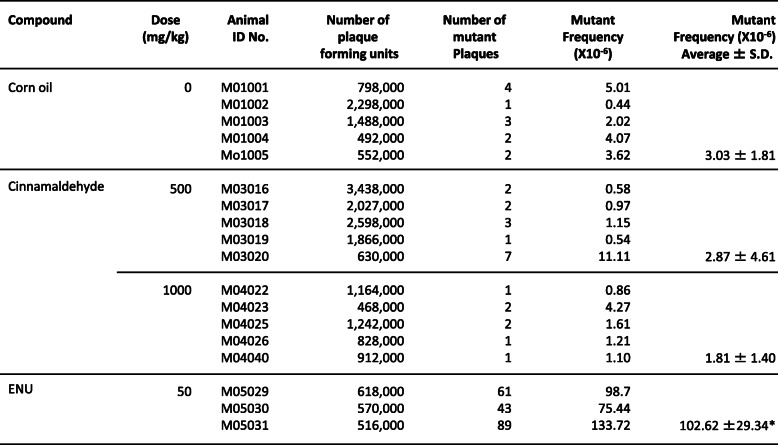
The results of TGR assay in small intestine of gpt delta mice after cinnamaldehyde treatment

Corn oil: Negative control (5 mL/kg)

ENU: positive control (N-ethyl-N-nitrosourea, 10 mL/kg*, i.p.*, dose once a day, for 5 days, expression period; 10 days)

*p < 0.05, significant difference from control (Welch’s I-test)

## Discussion

Of the 4500 types of food flavor currently registered worldwide, the flavors permitted for use differ among countries [24, 25] and not all flavors are guaranteed to be safe. If a chemical substance that is intentionally added to foods, such as a food flavor, is genotoxic and suspected to be carcinogenic, its use is typically banned across countries. Since it is difficult to perform carcinogenicity tests on many flavors due to the cost and requirement of large amount of flavor samples, the flavor chemicals permitted for use are usually determined by the results of genotoxicity tests.

The International Council for Harmonization of Pharmaceutical Regulations (ICH) M7 guideline, “Assessment and control of DNA reactive (mutagenic) impurities in pharmaceuticals to limit potential carcinogenic risk” issued in 2014 states that the assessment of genotoxicity for low-level chemicals such as pharmaceutical impurities should be conducted via Ames (mutagenicity) tests [26]. Other types of genotoxicants that are non-mutagenic typically have threshold mechanisms and usually do not pose carcinogenic risk in humans at the level ordinarily present as impurities. The guideline also recommends the use of QSAR analysis to predict the Ames test results as well as an in vivo TGR assay to follow-up on positive results from Ames tests. Similar to pharmaceutical impurities, food flavors are chemicals to which humans are exposed at low levels through food. Therefore, we assessed the mutagenicity of perillaldehyde and cinnamaldehyde according to the ICH-M7 approach.

Perillaldehyde and cinnamaldehyde have α,β-unsaturated aldehydes in their structure, which are a representative structural alerts for mutagenicity [27, 28]. Chemicals with an α,β-unsaturated aldehyde have electrophilicity that may interact with DNA. In addition to the carbon in the carbonylic functionality (1,2-addition), the β-carbon is positively polarized because of conjugation with the carbonyl group and becomes the preferred site of nucleophilic attack (1,4-addition) by the Michael reaction [29]. The first product of the 1,4-addition is a resonance-stabilized enolate ion.

In the present study, perillaldehyde did not show mutagenicity in the Ames test. Because the cyclic structure of perillaldehyde can inhibit enolate ion production and because β-carbon is probably inactive, perillaldehyde does not exhibit mutagenicity. Consistent with this result, perillaldehyde was previously reported to be negative in an Ames test [9, 30, 31]. Since this information is integrated into Derek Nexus and Case Ultra as knowledge, their QSAR predictions were “inactive” and “known negative,” respectively. We confirmed the negative result in the Ames test using a TGR assay with Muta™ Mice. Because gene mutations did not increase in the liver and glandular stomach of mice treated with perillaldehyde at the maximum dose tested, we concluded that perillaldehyde poses no risk of cancer-related mutagenicity.

Although perillaldehyde is a naturally occurring chemical used as a food flavoring worldwide and considered GRAS [6, 7], the European Food Safety Authority (EFSA) Panel on Food Contact Materials, Enzymes, Flavorings, and Processing Aids requested additional data related to the possible genotoxic potential of flavoring substances with α,β-unsaturated carbonyl structures including perillaldehyde. This request was made because α,β-unsaturated carbonyl compounds can react with nucleophilic sites in DNA through a 1,4-nucleophilic addition. In response to the EFSA request, perillaldehyde was assessed by Ames tests, in vitro micronucleus (MN) assays, and an in vitro HPRT mutation assay. In addition, in vivo MN and comet assays were also conducted in male rats. Results showed a statistically significant increase in revertant colony number according to the Ames test (TA98, −S9 mix), whereas the in vitro MN and HPRT mutation assays showed negative results. In in vivo MN and comet assays, there was no significant increase in MNs in the bone marrow of male rats following oral gavage administration of perillaldehyde doses up to 700 mg/kg/day; however, a small but statistically significant increase in comet tail intensity in the liver was observed at the highest dose (700 mg/kg/day). The study director reported that this small increase was within the distribution of historical negative control data and not biologically relevant; rather, it was most likely an artifact of the observed hepatic cytotoxicity. Therefore, the study director concluded that there was no genotoxic concern in vivo for perillaldehyde [32].

The results of these genotoxicity studies were reviewed by EFSA to determine whether perillaldehyde had genotoxic potential. Contrary to the conclusions stated in the study reported above, EFSA determined that the results of the in vitro HPRT mutation assay and in vivo comet assay were equivocal and positive, respectively. Based on concerns about genotoxicity in the liver, EFSA concluded that perillaldehyde was a potential safety concern as a flavoring substance [33, 34]. In response to the conclusion of EFSA, the Expert Panel of FEMA reviewed the newly available data and considered its interpretation relative to standard guidelines [35]. Ultimately, FEMA concluded that the results of the comet assay were consistent with the interpretation provided by the study director, i.e., that perillaldehyde does not appear to have any in vivo genotoxic potential [9]. Therefore, the genotoxic properties of perillaldehyde currently remain under dispute.

It may be difficult to end to the dispute between EFSA and FEMA with limited data because the battery of genotoxicity tests used for the assessment of genotoxic potential in perillaldehyde, conducted at the request of EFSA, is inappropriate and cannot be globally accepted. EFSA was initially concerned about in vitro mutagenicity in the Ames test and HPRT mutation assay. To confirm in vitro mutagenicity in vivo, it is necessary to conduct in vivo mutagenicity tests such as TGR assays. According to the ICH-S2 (R1) guideline (Guidance on Genotoxicity Testing and Data Interpretation for Pharmaceuticals Intended for Human Use), the comet assay is acceptable in follow-up studies to confirm the positive results of in vitro mammalian cell genotoxicity tests but not to confirm the positive results of Ames tests [36]. The ICH-M7 (R1) guideline recommends the TGR assay as a follow-up test if an impurity in pharmaceuticals produces positive results in an Ames test [26]. Therefore, it is internationally agreed that a TGR assay is essential for confirming Ames mutagenicity. In the current study, we clearly demonstrated that perillaldehyde was negative for mutagenicity in both an Ames test and TGR assay. It is unclear why our Ames test result was negative whereas EFSA’s result was positive. The purity of perillaldehyde used in our study was 97.3% but in the EFSA study it was 91.9–94.2%. In addition, the Ames test by EFSA showed a clear increase in mutants only at high doses (> 1000 μg/plate). This suggests that a small amount of impurity in perillaldehyde in the EFSA study may have produced the Ames mutagenicity. Regardless, there are no concerns about the in vivo mutagenicity of perillaldehyde because of the negative result shown in our TGR assay. We hope that EFSA will review our current study and reassess the mutagenicity of perillaldehyde in the near future.

EFSA determined that cinnamaldehyde lacks direct mutagenic and genotoxic activity [37, 38], although positive results have been recorded in some in vitro and in vivo genotoxicity tests, including Ames tests, and cinnamaldehyde has α,β-unsaturated aldehydes in its structure [10, 11, 30, 31]. Ishidate et al. reported a positive result in an Ames test for cinnamaldehyde in the TA100 strain [30, 31]; however, only borderline mutagenicity was observed in the absence of S9-mix, with the revertant frequency slightly more than twice the spontaneous frequency. In the present study, cinnamaldehyde showed a similar response, i.e., the maximum revertant frequency in the TA100 strain in the absence of S9-mix was slightly more than twice that in the negative control. Trans-cinnamaldehyde (cas# 104–55-2), 4′-methoxy cinnamaldehyde (cas#1963-36-6), and benzalacetone (4-phenyl − 3-buten-2-one; cas# 122–57-6), which are cinnamaldehyde-related flavor chemicals, have also shown positive results in Ames tests [39]. We concluded that cinnamaldehyde and its derivatives are mutagenic in vitro, given that they show reproducible Ames mutagenicity and have α,β-unsaturated aldehydes. However, the TGR assay employed in the present study clearly demonstrates that there is no concern about in vivo mutagenicity from cinnamaldehyde. α,β-Unsaturated aldehydes are converted to less electrophilic molecules via three pathways in vivo: oxidation, conjugation with glutathione, and reduction. The detoxification efficiency and reaction efficiency with DNA vary depending on the structure [40]. Kiwamoto et al. demonstrated that although cinnamaldehyde induces a higher DNA adduct level than other α,β-unsaturated aldehydes, this level is three orders of magnitude lower than the natural background levels of structurally similar DNA adducts observed in the human liver, i.e., the observed level does not show mutagenicity [41]. Indeed, most α,β-unsaturated aldehydes may be of no concern in terms of mutagenicity and carcinogenicity in vivo.

In conclusion, the present study clearly demonstrates that perillaldehyde and cinnamaldehyde do not produce in vivo mutagenicity when administered at doses up to 1000 mg/kg/day in mice; however, cinnamaldehyde is mutagenic in vitro.

## Data Availability

All generated data are included in this manuscript.

## Abbreviations

QSAR: Quantitative structure–activity relationship
TGR: Transgenic rodent gene mutation
GRAS: Generally recognized as safe
FEMA: Flavor and Extract Manufactures Association
EFSA: European Food Safety Authority
GLP: Good laboratory practice
OECD: Organization of Economic Co-operation and Development
MF: Mutant frequency
ICH: International Council for Harmonization of Pharmaceutical Regulations
